# Synthesis and Characterization of Organosoluble, Thermal Stable and Hydrophobic Polyimides Derived from 4-(4-(1-Pyrrolidinyl)phenyl)-2,6-bis(4-(4-aminophenoxy)phenyl)pyridine

**DOI:** 10.3390/polym9100484

**Published:** 2017-10-03

**Authors:** Xiaohua Huang, Beicai Chen, Mei Mei, Hua Li, Chanjuan Liu, Chun Wei

**Affiliations:** Key Laboratory of New Processing Technology for Nonferrous Metal & Materials, Ministry of Education, and School of Material Science and Engineering, Guilin University of Technology, Guilin 541004, China; chenbeicai1101@gmail.com (B.C.); mm15964962995@gmail.com (M.M.); lihua15909149555@gmail.com (H.L.); 1986024@glut.edu.cn (C.W.)

**Keywords:** polyimides, thermal stability, solubility, hydrophobicity

## Abstract

A novel aromatic diamine monomer, 4-(4-(1-pyrrolidinyl)phenyl)-2,6-bis(4-(4-aminophenoxy)phenyl)pyridine (PPAPP) containing pyridine rings, pyrrolidine groups, and ether linkages, was successfully synthesized using 4-hydroxyacetophenone and 1-chloro-4-nitrobenzene as starting materials by three-step reactions, and then used to synthesize a series of polyimides by polycondensation with various aromatic dianhydrides via a two-step method. The structure of PPAPP was characterized by NMR, FT-IR, and mass spectrometry analysis methods. These polymers showed good solubility in common organic solvents (e.g., NMP, DMF, DMSO, and DMAc) at room temperature or on heating. Moreover, they presented a high thermal stability with the glass transition temperature (*T*_g_s) exceeding 316 °C, as well as the temperature of 10% weight loss ranged from 552 to 580 °C with more than 67% residue at 800 °C under nitrogen. Furthermore, they also exhibited excellent hydrophobicity with a contact angle in the range of 85.6° to 97.7°, and the results of Wide-Angle X-ray Diffraction (WAXD) indicated that all of the polymers revealed an amorphous structure.

## 1. Introduction

Aromatic polyimides (PIs) are a class of macromolecular high-performance polymers possessing the imide rings and aromatic groups in the main chains [1,2,3,4,5]. They have been applied in the fields of electronic devices, microelectronic packaging, gas separation, optoelectronic, and automobile industries due to their unique heat resistance, superior chemical stability, special physical, and their mechanical properties [6,7,8,9,10,11,12]. However, although possessing the superior comprehensive properties mentioned above, most of these polymers show some serious drawbacks. For example, poor solubility in most organic solvents, high softening and meting temperatures, and deeply color that often limit their widespread application in various advanced technologies [13,14,15,16,17,18,19]. Thus, many efforts have been devoted to improving the solubility, processability, and highly optical transparent of PIs by the incorporation of flexible or twist linkages [20,21,22,23], asymmetric noncoplanar units [24,25,26], bulky pendant groups [27,28,29], and fluorinated substituents [30,31,32,33,34] into the polymer backbones.

However, these strategies mentioned above usually suffer from compromise between the thermal properties and the solubility of PIs, and it is because of the same structural characteristics that increase one will also reduce the other. Therefore, how to balance between thermal properties and solubility of PIs becomes an important challenge. Among these approaches, the introduction of pyridine units into the main chain is an effective way to improve the thermal properties of polymers [35,36,37,38,39]. As is well known, pyridine belongs to a class of hetero-aromatic nitrogen compound with greater rigidity and stronger polarization. The advantages of pyridine ring in different heterocycles come from its molecular symmetry and aromatic high thermal stability [40]. In addition, the presence of nitrogen atoms in pyridine rings can generate polarized bonds, which increase the solubility of polymers due to the intermolecular dipole-dipole interactions in a polymer-solvent system [41,42,43]. For example, PIs derived from 2-(5-(3,5-diaminophenyl)-1,3,4-oxadiazole-2-yl)pyridine [44] and 1,3-bis(5-amino-2-pyridinoxy)benzene [45] have all have been demonstrated to own high solubility and thermal properties. Li et al. [46] reported a class of cardo-type polyimides with cyclopentyl-containing alicyclic units as side groups. These polyimides presented good solubility, high thermal stability and optical transparency, ecellent mechanical properties, low water absorption, and a dielectric constant.

In this article, based on the previous research progress, a novel aromatic diamine 4-(4-(1-pyrrolidinyl)phenyl)-2,6-bis(4-(4-aminophenoxy)phenyl)pyridine (PPAPP) was synthesized, and then used to prepare a series of PIs containing pyridine units, alicyclic pyrrolidine, and ether groups in the polymer chain. Making full use of their different groups synergies, a series of PI materials having excellent overall performance were hoped to be designed and synthesized. Simultaneously, the solubility, thermal stability, hydrophobicity, and aggregate structure of PIs were also investigated.

## 2. Experimental Section

### 2.1. Materials

4-Hydroxyacetophenone (Accela ChemBio Co., Ltd., Shanghai, China), 1-chloro-4-nitrobenzene (Shanghai Darui Chemical Co., Ltd., Shanghai, China), 4-(1-pyrrolidinyl)benzaldehyde (Accela ChemBio Co., Ltd.), hydrazine hydrate (Sinopharm Chemical Reagent Co., Ltd., Shanghai, China), palladium 10% on carbon (wetted with ca. 55% water) (TCI). Pyromellitic dianhydride (PMDA, Sinopharm Chemical Reagent Co., Ltd.), biphenyl tetracarboxylic dianhydride (BPDA) and oxydiphtahalic anhydride (ODPA) (Changzhou Linchuan Chemical Co., Ltd., Changzhou, China), benzophenone tetracarboxylic dianhydride (BTDA), and 4,4′-(hexafluoroisopropylidene) diphthalic anhydride (6FDA) (Tokyo Chemical Industry Co., Ltd., Tokyo, Japan) were recrystallized from acetic anhydride and then dried in vacuum at 120 °C overnight before use. *N*,*N*-Dimethylformamide (DMF) and *N*-methyl-2-pyrrolidone (NMP) (Shanghai Guoyao Chemical Co., Ltd., Shanghai, China) were purified by vacuum distillation over calcium hydride prior to use. All of the other solvents were of analytical grade and were used without further purification.

### 2.2. Preparation of Monomer

#### 2.2.1. Synthesis of 4-(4-Nitrophenoxy)acetophenone (NPAP)

In a 500 mL three-necked round bottom flask equipped with magnetic force stirrer, reflux condenser, and nitrogen inlet, 4-hydroxyacetophenone (40.00 g, 0.29 mol), 1-chloro-4-nitrobenzene (46.28 g, 0.29 mol), potassium carbonate (85.27 g, 0.62 mol), and 300 mL DMF was refluxed for 6 h at 145 °C. After the reaction was terminated, the reaction mixture was cooled to 60 °C, and then poured into ice water with small amount of NaCl. The precipitate was filtered to give a yellow product. Finally, the crude product was recrystallized from ethanol and ethyl acetate (*V*:*V* = 1:1) mixture solution to obtain white pure product 4-(4-nitrophenoxy) acetophenone (NPAP). Yield: 65%. ^1^H NMR (500 MHz, DMSO, δ, ppm): 8.25 (d, 2H), 8.04 (d, 2H, *J* = 8.4 Hz), 7.22 (t, 4H, *J* = 9.8 Hz), 2.57 (s, 3H, –CH_3_). ^13^C NMR (500 MHz, DMSO, δ, ppm): 197.32, 162.14, 159.27, 143.69, 134.19, 131.65, 126.97, 120.31, 119.38, 27.39. FT-IR (KBr, cm^−1^): 3109, 3073 (–CH_3_), 1681 (C=O), 1507, 1347 (–NO_2_), 1168 (C–O–C). m.p. 83 °C (by DSC at a scan rate of 5 K/min).

#### 2.2.2. Synthesis of 4-(4-(1-Pyrrolidinyl)phenyl)-2,6-bis(4-(4-nitrophenoxy)phenyl)pyridine (PPNPP)

10 g (0.057 mol) of 4-(1-pyrrolidinyl)benzaldehyde, 29.36 g, (0.11 mol) of NPAP, 52.78 g of ammonium acetate and 130 mL acetic acid were placed into a 500 mL three-necked flask equipped with a magnetic force stirrer, reflux condenser, and nitrogen inlet. The mixture was refluxed with stirring for 6 h at 125 °C. After the reaction mixture was cooled to room temperature, and then concentrated in a vacuum to remove excess acetic acid, the yellow crude power PPNPP was obtained under vacuum drying at 120 °C for 12 h. The pure PPNPP was acquired after the precipitate purified by recrystallization in DMF and vacuum drier at 120 °C. Yield: 61%. ^1^H NMR (500 MHz, DMSO, δ, ppm): 8.46 (d, *J* = 8.7 Hz, 4H), 8.31 (d, *J* = 9.0 Hz, 4H), 8.17 (s, 2H), 7.98 (d, *J* = 8.8 Hz, 2H), 7.36 (d, *J* = 8.7 Hz, 4H), 7.25 (d, *J* = 9.5 Hz, 2H), 6.69 (d, *J* = 8.9 Hz, 2H), 3.35 (d, *J* = 24.5 Hz, 6H), 2.02 (m, 4H). FT-IR (KBr, cm^−1^): 1596, 1439 (pyridine), 1514, 1342 (–NO_2_), 3100~3000 (–CH_2_–, pyrrolidine), 1165 (C–O–C). m.p. 267 °C (by DSC at a scan rate of 5 K/min).

#### 2.2.3. Synthesis of 4-(4-(1-Pyrrolidinyl)phenyl)-2,6-bis(4-(4-aminophenoxy)phenyl)pyridine (PPAPP)

To a 500 mL three-necked round-bottomed flask equipped with a magnetic force stirrer, reflux condenser and nitrogen inlet, PPNPP (16.62 g, 0.026 mol), Pd/C (3.5 g), and 300 mL of ethanol absolute, then the mixture solution was heated to 85 °C for 30 min. After 65 mL hydrazine monohydrate was dropped for 1 h, the reaction mixture was stirred for another 8 h. 80 mL extra tetrahydrofuran was added before the end of the reaction, and then refluxed for another 10 min. The mixture solution was followed by hot filtration to remove excess Pd/C, evaporated and dried under vacuum at 120 °C for 12 h to get crude product PPAPP. The pure PPAPP was obtained by silica gel column chromatography (*V*_Dichloromethane_:*V*_Ethyl acetate_ = 30:2, Rf = 0.35) and vacuum drier at 120 °C. Yield: 90%. ^1^H NMR (500 MHz, DMSO, δ, ppm): 8.23 (d, 4H, *J* = 8.5 Hz), 7.95 (s, 2H), 7.87 (d, 2H, *J* = 8.5 Hz), 7.00 (d, 4H, *J* = 8.5 Hz), 6.87 (d, 4H, *J* = 8.5 Hz), 6.65 (m, 6H), 5.05 (s, 4H, –NH_2_), 3.28 (t, 4H, *J* = 6 Hz), 1.96 (m, 4H). ^13^C NMR (500 MHz, DMSO, δ, ppm) 160.24, 156.15, 149.77, 148.84, 145.90, 145.82, 133.40, 128.81, 128.31, 121.48, 116.85, 115.53, 114.11, 112.33, 47.73, 43.45, 25.43. FT-IR (KBr, cm^−1^): 3300~3500 (–NH_2_), 3100~3000 (–CH_2_–, pyrrolidine), 1168 (C–O–C), 1595, 1438 (pyridine). MS (*m*/*z*): 591.27 ([M + H]^+^). m.p. 181 °C (by DSC at a scan rate of 5 K/min).

### 2.3. Preparation of Poly(Amic Acid) (PAA)

The polyimides were synthesized from PPAPP and various dianhydrides via a two-step method. The first step, a typical example of polymerization was as follows. The synthesis of PI-1 (PPAPP and PMDA) was used as an example to illustrate the general synthetic route used to produce the polyimides. A mixture of 0.6176 g (0.001042 mol) PPAPP and 5 mL of dry NMP in 25 mL flask, 0.2273 g (0.001042 mol) of PMDA was added in two portions. Therefore, the solid content of the solution was maintained approximately 20 wt %. The mixture was stirred at room temperature for 24 h to afford highly viscous poly(amic acid)s (PAA) solution. Simultaneously, the other PAA solutions were prepared by similar procedure.

### 2.4. Preparation of PI Films

The second step, the PAA obtained above, the PAA precursors obtained above, were then transformed into polyimide by the thermal imidization process. For this method, the PAA solution was poured onto a clean glass substrate, and then placed at 80 °C for 12 h to slowly to release the most casting solvent. The semidry PAA was further dried by programmed heating at 150 °C, 200 °C, 250 °C, and 300 °C, for each 1 h, and then the PI film was peeled off from the glass substrate by immersion in water. Finally, PI-1 (PPAPP–PMDA) film was obtained after drying at 150 °C for 12 h before being measured. The thermal imidization method was also adopted by the above method to obtain other PI films, which named PI-2 (PPAPP–BPDA), PI-3 (PPAPP–ODPA), PI-4 (PPAPP–BTDA), and PI-5 (PPAPP–6FDA).

### 2.5. Characterization Methods

#### 2.5.1. Fourier Transform Infrared (FT-IR)

Fourier Transform Infrared (FT-IR) of monomer and polymer were tested on Thermo Nexus 470 FT-IR spectrometer (Thermo Nicolet Corporation, Middletown, VA, USA).

#### 2.5.2. Nuclear Magnetic Resonance (NMR)

^1^H NMR and ^13^C NMR of monomer were measured on an Avance AV 500 instrument (Bruker, Fällanden, Switzerland) using deuterated dimethylsulfoxide (DMSO-*d*_6_) as solvents.

#### 2.5.3. Mass Spectrometry (MS)

Mass spectrometry was recorded on a Elementar Vario EL III/Isoprime (Elementar, Langenselbold, Germany).

#### 2.5.4. Thermo-Gravimetric Analysis (TGA)

The thermal stability of films was performed on a TGA Q500 analyzer (TA Instruments, Brussels, Belgium). Films were dried at 150 °C for 30 min and then heated from 50 °C to 800 °C at a heating rate of 10 °C/min in nitrogen.

#### 2.5.5. Differential Scanning Calorimetry (DSC)

The glass transition temperatures (*T*_g_) of films were recorded on a DSC-204 phoenix thermal analyzer (Netzsch, Wittelsbacherstr, Germany) at a heating rate of 10 °C/min in nitrogen atmosphere.

#### 2.5.6. Solubility Analysis

10 mg of polymer was dissolved in 1 mL organic solvent and observed the dissolving state of polymer at room temperature or heating conditions.

#### 2.5.7. The Contact Angle

Surface hydrophobicity was carried out on the film for water was tested by a contact angle goniometer instrument (JY-PHb, Changsha, China) with 40 ± 2% relative humidity at the room temperature.

#### 2.5.8. The Wide-Angle X-ray Diffraction (WAXD)

A crystallographic study of films was measured at room temperature (about 25 °C), on an X’Pert PRO X-ray diffractometer (PANalytical B.V., Almelo, The Netherlands). The data was taken from 10° to 40° (2θ values) with Cu/Kα radiation (λ = 0.154 nm, operating at 40 kV and 40 mA).

#### 2.5.9. Inherent Viscosities

Inherent viscosities of PAA were tested by Ubbelohde viscometer. It was measured at a concentration of 0.5 g/dL in NMP at 25 °C.

## 3. Results and Discussion

### 3.1. Monomer Synthesis

**Scheme 1:**
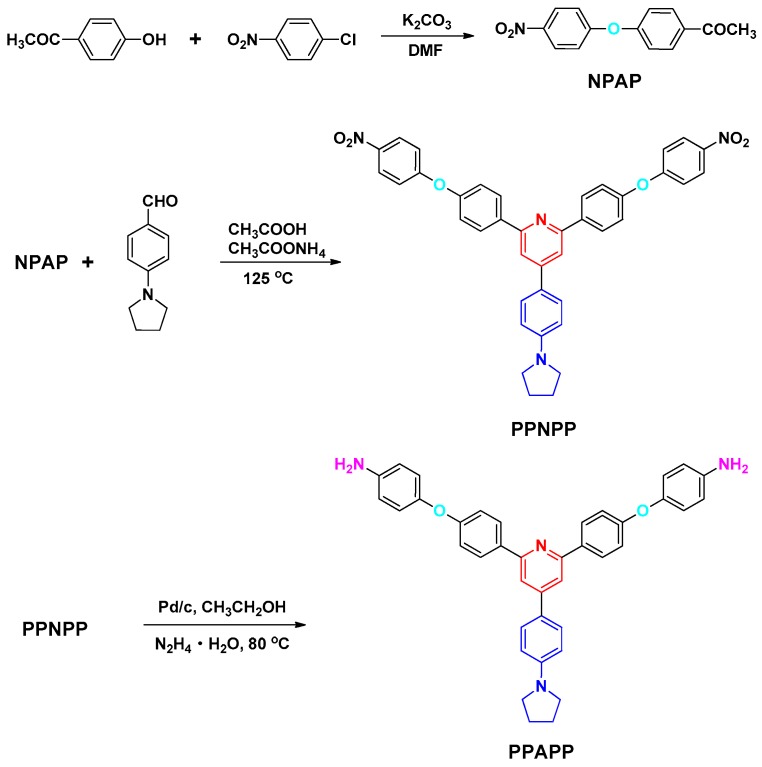
Synthesis of diamine monomer 4-(4-(1-pyrrolidinyl)phenyl)-2,6-bis(4-(4-aminophenoxy)phenyl)pyridine (PPAPP).

PPAPP was synthesized by a three-step synthetic route, as illustrated in Scheme 1. Firstly, the 4-hydroxyacetophenone reacted with 1-chloro-4-nitrobenzene in DMF to get the intermediate compound NPAP. Secondly, the dinitro compound PPNPP was obtained via modified Chichibabin reaction, which reported the best way to get pyridine rings from NPAP and 4-(1-pyrrolidinyl)benzaldehyde. Finally, the PPAPP was obtained the reduction of PPNPP with hydrazine monohydrate and Pd/C catalyst.

**Figure 1:**
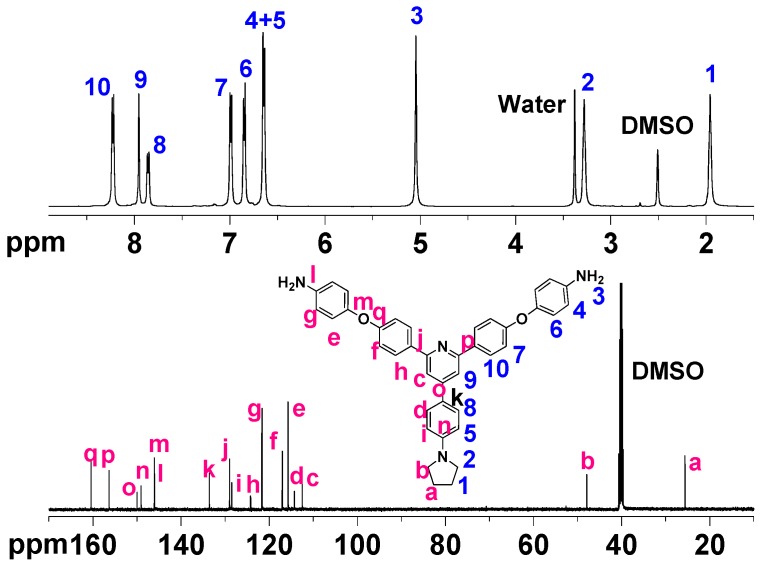
^1^H (**up**) and ^13^C NMR (**down**) spectra of PPAPP.

As shown in Figure S1, the FT-IR spectrum of NPAP showed the characteristic absorptions of the -NO_2_ group appeared at 1348 cm^−1^, the C–O–C stretching bands appeared at 1167 cm^−1^, the C=O characteristic stretching absorptions was also measured at 1682 cm^−1^, and the –CH_3_ stretching bands appeared at 3109–3073 cm^−1^. In Figure S2, FT-IR spectra of PPNPP showed characteristic bands of pyridine at 1596 cm^−1^, 1439 cm^−1^, methylene groups from pyrrolidine at 3100–3000 cm^−1^, and the characteristic stretching of the C=O disappeared after reduction. It can be seen from Figure S3, that the characteristic absorptions of the –NH_2_ group appeared at 3300–3500 cm^−1^, the C–O–C stretching bands appeared at 1168 cm^−1^, and pyridine ring characteristic stretching absorptions was also measured at 1595 cm^−1^. As shown in Figure S4, ^1^H NMR spectrum of NPAP showed the characteristic aromatic peaks at 7.20–8.30 ppm and –CH_3_ protons at 2.56 ppm. Furthermore, in the ^13^C spectrum 8 resonances assigned to the aromatic carbon atoms, can be revealed in the 170–100 ppm range; the CO group at 197.49 ppm, and the methyl group at 27.06 ppm. In Figure S5, the ^1^H NMR spectra of PPNPP showed the aromatic protons are appeared in the range of 6.67–8.40 ppm, and pyrrolidine protons (–CH_2_–) peaks at 3.35, 1.99 ppm. The ^1^H and ^13^C NMR spectra of PPAPP were showed in Figure 1. The signal at 5.05 ppm in the ^1^H NMR spectrum of PPAPP corresponds to the –NH_2_ groups and resonances between 6.65–8.23 ppm are referred to as the aromatic protons. The ^13^C NMR spectrum also showed that all of the carbon atoms resonated in the vicinity of 25.43–160.24 ppm, and also all of the spectrum was well consistent with the proposed molecular structure. In addition, in Figure S6, MS spectroscopic analysis of PPAPP was in good agreement with the calculated results.

### 3.2. Polymer Synthesis

**Scheme 2:**
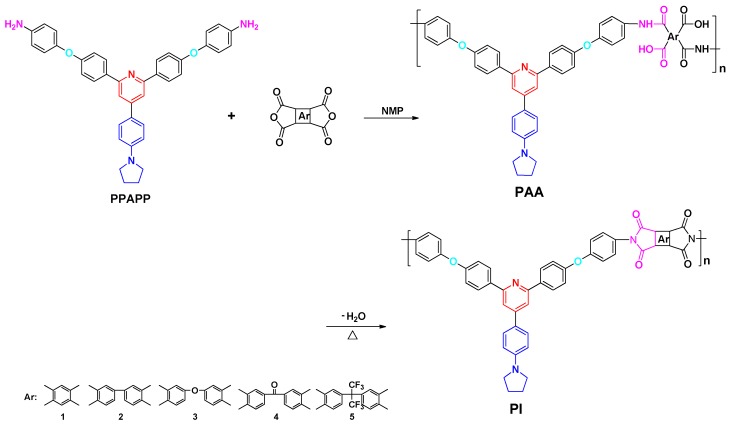
Synthesis of polyimides.

As shown in Scheme 2, PIs were prepared from diamines PPAPP and five kinds of dianhydrides by a conventional two-step synthetic method. Firstly, PPAPP was dissolved in a measured amount of purified NMP, and then the equimolar amount of dianhydride monomer was added slowly in two portions. Subsequently, the solution was stirred 24 h at room temperature, forming viscous poly(amic acid) (PAA) solutions. A series of PIs were obtained by sequential thermal imidization method. The inherent viscosities of PAA were tested by Ubbelohde viscometer, and measured at concentration of 0.5 g/dL in NMP at 25 °C. As shown in Table 1, PAAs have moderate inherent viscosities in the range of 0.36–0.67 dL/g in NMP, which also indicated that polymers have high molecular weight.

**Figure 2:**
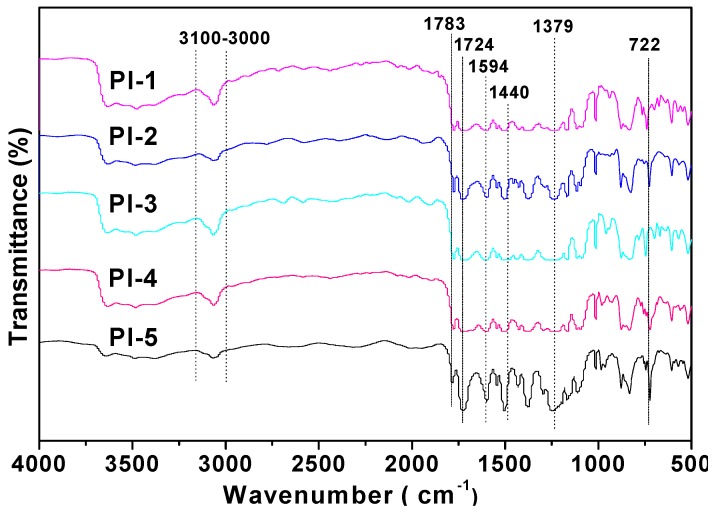
Fourier Transform Infrared (FT-IR) spectra of polyimide filmes.

The chemical structures of PIs were characterized by FT-IR spectroscopy, as shown in Figure 2. The FT-IR spectra showed the characteristic absorption at 1783 cm^−1^ (asymmetrical stretching) and 1724 cm^−1^ (symmetrical stretching), C–N stretching at 1379 cm^−1^, and imide ring deformation at 722 cm^−1^. The characteristic absorptions of pyridine was appeared in 1594 and 1440 cm^−1^. The stretching absorption of methylene from pyrrolidine were also observed in the range of 3100–3000 cm^−1^. Obviously, the disappearance of the characteristic absorption peaks of –NH_2_ group near 3200–3350 cm^−1^ indicated the complete formation of imide structure.

### 3.3. Thermal Properties

**Figure 3:**
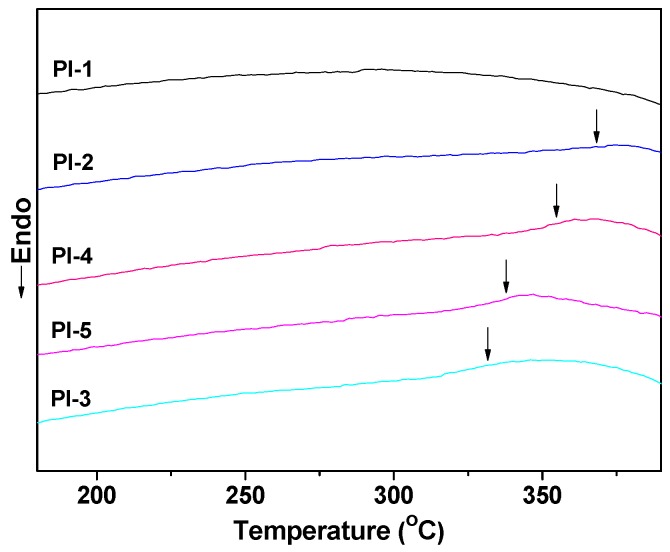
Differential Scanning Calorimetry (DSC) curves of polyimides.

The thermal properties of PIs were evaluated by DSC and TGA at heating rate of 10 °C/min under N_2_ atmosphere. DSC curves of PIs are shown in Figure 3, and the glass transition temperatures (*T*_g_s) obtained from the second heating curve of DSC are outlined in Table 1. The *T*_g_ values of these polymers all exceeded 316 °C, and just as expected, the different values of *T*_g_s are related to the structure of the dianhydride component. In general, *T*_g_s will decrease with an increasing flexibility of the structure of dianhydride. On the contrary, with the increase in rigidity of dianhydride structure, *T*_g_s of polymers will be raised. Obviously, PI-1 showed no *T*_g_ in Figure 3, and it may be due to the presence of rigid pyromellitic units in the polymer chain, which limited the free rotation of molecular chain greatly and led to the *T*_g_ higher than its decomposition temperature. The *T*_g_ values of PI-2–PI-5 were in the range of 316–359 °C. PI-2 presented higher *T*_g_ (359 °C) due to the introduction of relatively rigid biphenylic units. However, it was found that PI-3 showed the minimum *T*_g_ value (316 °C) than that of other polymers, which might be attributed to the incorporation of flexible ether linkages moieties from ODPA in the polymer backbone.

**Figure 4:**
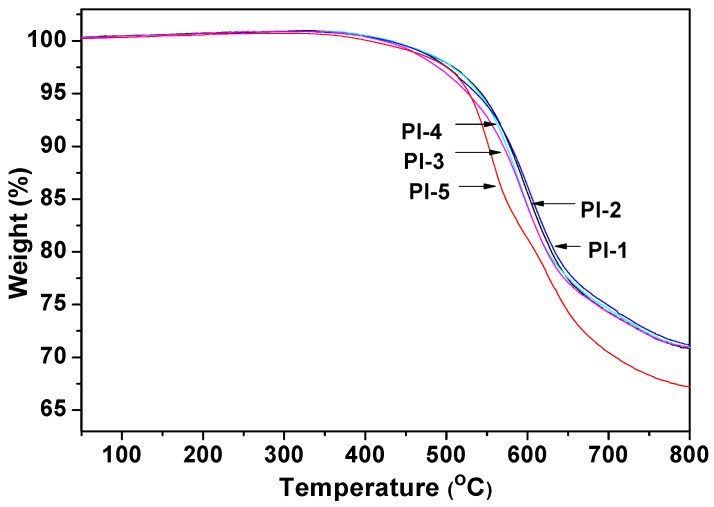
Thermo-Gravimetric Analysis (TGA) curves of polyimides.

TGA curves for PIs were shown in Figure 4. The onset decomposition temperatures (*T*_d_), the decomposition temperatures of 5% and 10% weight loss, and char yield were recorded from the original TGA thermal analysis diagram, and the results were summarized in Table 2. All of these polymers exhibited excellent thermal stability with the temperature of 5% and 10% weight loss being in range of 527–543 °C and 552–580 °C in nitrogen atmosphere, respectively, indicating their different thermal stability. In addition, the residual weight retentions at 800 °C for the resulting PIs were in the range of 67–71%. The outstanding thermal stability of PIs was displayed, and it is due to the presence of rigid moieties, such as stiff imide rings, pyridine groups, and bulky pendent groups in polymer structure [47,48]. In need of special note is that the introduction of heterocycle pyridine and pyrrolidine groups in the main chains endowed an increase in the thermal stability of polymers. Meanwhile, the high heat resisitance of polyimids also have been endowed because of its polarizability that resulted from the nitrogen atom in the pyridine and alicyclic pyrrolidine rings [2].

### 3.4. Solubility Properties

The solubility behavior of PIs was tested with a polymer concentration of 10 mg/mL at room temperature or on heating in various common organic solvents, and the results were summarized in Table 2. All of the polyimdes presented a good solubility in polar aprotic solvents, such as DMF, DMAc, DMSO, and NMP, and even they could be partly dissolved in the low boiling point solvent, such as CHCl_3_ and THF. In general, the solubility of polymers was closely connected with their molecular structure. For example, increasing the molecular chain distance or the space free volume and decreasing the interaction and packing density of polymer chain were considered effective ways to improve polymer solubility. Therefore, the introduction of bulky pendant groups, flexible ether linkages, and non-coplanar structures into the polymer main chain would inevitably promote polymer solubility. In Table 2, obviously, the solubility of PIs was found to increase in the following order PI-5 (PPAPP-6FDA) > PI-3 (PPAPP-ODPA) > PI-4 (PPAPP-BTDA) > PI-2 (PPAPP-BPDA) > PI-1 (PPAPP-PMDA). PI-1 based on PMDA showed soluble in NMP on heating, partially soluble in DMF, and DMAc on heating due to the presence of rigid pyromellitic in the main chains, which made it difficult for the solvent to attack. PI-2 derived from BPDA showed to be soluble in DMF and NMP on heating, partially soluble in DMSO and DMAc on heating, and it is because of the introduction of relatively rigid biphenylic moieties in the polymer chains. However, surprisingly enough, PI-3 and PI-5 displayed excellent solubility in DMF, DMAc, DMSO, and NMP at room temperature. Moreover, in the low boiling point solvent, PI-3 showed soluble in CHCl_3_ and THF on heating due to the incorporation of flexible ether linkages in the polymer backbones. Furthermore, PI-5 revealed to be soluble in CHCl_3_ and THF at room temperature, partially soluble in CH_2_Cl_2_ on heating, and it may be due to the introduction of the bulky pendent –C(CF_3_)_2_– groups in the polymer structure. It is obvious that the presence of flexible ether linkages and bulky pendent groups with alicyclic pyrrolidine as side groups in the main chains were all beneficial to disturb the packing of molecules and make the solvent molecule penetrate easily, and then promote the solubility of the polymer. Moreover, the incorporation of nitrogen atoms from pyridine and pyrrolidine rings can generate polarized bonds, which can improve the solubility of polyimides due to the intermolecular dipole-dipole interactions in polymer–solvent system [42]. In addition, the dissolution characteristics of polymers in low-boiling solvent was very convenient for processing and forming them into desired products by solution casting method at lower temperatures.

### 3.5. Hydrophobic Properties

**Figure 5:**
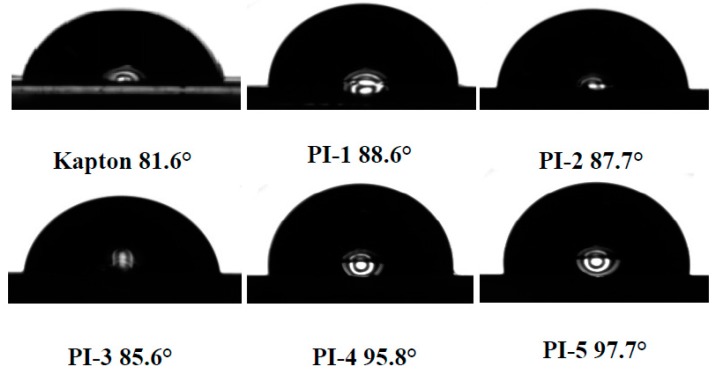
The contact angle of Kapton and Polyimide films.

As shown in Figure 5, the hydrophobic property of PI films were evaluated by measuring their contact angles, and the results were listed in Table 1. In general, the higher contact angle of polymer has, the better hydrophobicity of polymer will exhibit. All the polymers, presented excellent hydropobicity with the contact angles in the range of 85.6°–97.7°, and higher than that of commercial Kapton (81.6°) [49,50]. This fact is attributed to the presence of hydrophobic pyridine and bulky pendent groups in the polymer backbones. Furthermore, the presence of heterocyclic units of polyimides, such as pyridine and pyrrolidine rings in the polymer structures, is more resistant to hydrolysis and nucleophilic attack than that of imide rings, that is to say, which improve indirectly the hydrolytic stability of polyimides [2]. Obviously, PI-5 based on 6FDA showed the highest hydrophobic value (97.7°), and that is because the incorporation of fluorine-containing hexafluoroisopropylidene groups in the polymer chain decreased the surface energy of the polymer, and then displayed the best hydrophobicity. However, the contact angle of PI-3 originated from ODPA revealed the lowest hydrophobic value (85.6°), and it may be due to the ether linkage from ODPA forming hydrogen bonds with water.

### 3.6. WAXD Data

**Figure 6:**
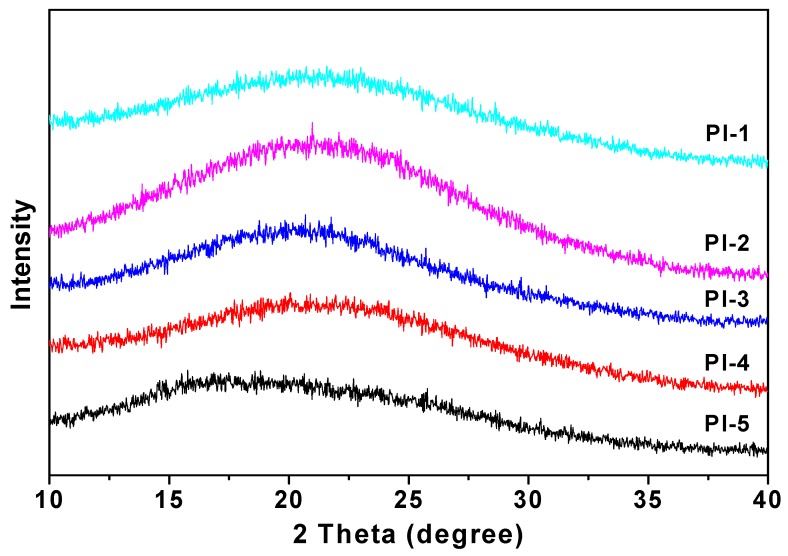
WAXD patterns of the polyimides.

The crystallinities of polymers were investigated by Wide angle X ray diffraction (WAXD) with graphite monochromatized CuKα ration and 2θ ranging from 10° to 40° at room temperature. As shown in Figure 6, the representative X-ray diffractograms indicated that all of the PIs are an amorphous pattern with a wide dispersion peak, and it could be mainly explained by incorporation of bulky pendent groups, flexible linkages, and co-planar structures in the polymer chains, and then led to increasing the spacing between molecular chains, decreasing the intermolecular interactions, and the packing density of the polymer backbone, and resulting in the formation of amorphous structures. Moreover, no melting peak was observed except the *T*_g_ in Figure 3, which was also in accordance with the analysis results of WAXD. In addtion, the amorphous nature of PIs also reflected their good solubility in organic solvents.

## 4. Conclusions

In this article, a series of novel polyimides containing pyridine rings, alicyclic pyrrolidine groups, and flexible ether linkages were prepared from PPAPP and commercially aromatic dianhydrides via two-step procedure. All of these polymers revealed a high thermal stability, an excellent solubility, and outstanding hydrophobicity. Moreover, they could be used as a class of high performance polymer materials for applications in the fields of electronic packaging materials, gas separation membrane, microelectronics, photoelectrons, and so on.

## Figures and Tables

**Table 1:** Thermal properties, viscosity and contact angle of polyimides.

Polymers	*T*_g_ (°C) ^a^	*T*_d_ (°C) ^b^	*T*_5%_ (°C) ^c^	*T*_10%_ (°C) ^c^	*R*_w_ (%) ^d^	*Η* (dL/g)	θ_w_ (°)
PI-1	--	534	537	580	70.8	0.37	88.6
PI-2	359	533	540	574	70.9	0.36	87.7
PI-3	316	527	527	570	71.2	0.41	85.6
PI-4	348	536	543	578	71.0	0.63	95.8
PI-5	322	517	528	552	67.2	0.67	97.7

^a^ *T*_g_: Midpoint temperature of baseline shift on the second DSC heating trace (rate of 10 °C/min) of the sample after quenching from 400 °C in N_2_; ^b^ *T*_d_: onset decomposition temperature, recorded via TGA at a heating rate of 10 °C/min; ^c^ Temperature of 5% and 10% weight loss recorded via TGA at a heating rate of 10 °C/min. ^d^ Char Yield (wt %) at 800 °C in nitrogen.

**Table 2:** Solubility of polyimides.

Polymers	Solvent ^a^							
	DMF ^b^	DMAC ^b^	DMSO ^b^	NMP ^b^	CHCl_3_	THF ^b^	CH_2_Cl_2_	EA
PI-1	+−	−−	+−	+	−−	−−	−−	−−
PI-2	+	+−	+−	+	−−	−−	−−	−−
PI-3	++	++	++	++	+	+	−−	−−
PI-4	++	+	+	++	+	+−	−−	−−
PI-5	++	++	++	++	++	++	+−	−−

^a^ Solubility was tested with a polymer concentration of 10 mg/mL in solvent with stirring; ^b^ THF (tetr**a**hydrofuran); EA (ethyl alcohol); soluble at room temperature (++); soluble on heating (+); partially soluble on heating (+−); insoluble (−−).

## Supplementary Materials

The following are available online at www.mdpi.com/2073-4360/9/10/484/s1, Figure S1: FT-IR spectra of compound NPAP; Figure S2: FT-IR spectra of compound PPNPP; Figure S3: FT-IR spectra of diamine PPAPP; Figure S4: ^1^H and ^13^C NMR spectra of compound NPAP; Figure S5: ^1^H NMR spectra of compound PPNPP; Figure S6: MS spectra of diamine PPAPP.

## References

[1] Ding M.X. (2007). Isomeric polyimides. Prog. Polym. Sci..

[2] Liaw D.J., Wang K.L., Huang Y.C., Lee K.R., Lai J.Y., Ha C.S. (2012). Advanced polyimide materials: Syntheses, physical properties and applications. Prog. Polym. Sci..

[3] Ghosh A., Sen S.K., Banerjee S., Voit B. (2012). ChemInform abstract: solubility improvements in aromatic polyimides by macromolecular engineering. RSC Adv..

[4] Yi L., Huang W., Yan D.Y. (2017). Soluble aromatic polyimides with high glass transition temperature from benzidine containing tert-butyl groups. J. Polym. Sci. A.

[5] Vanherck K., Koeckelberghs G., Vankelecom I.F.J. (2013). Crosslinking polyimides for membrane applications: A review. Prog. Polym. Sci..

[6] Ghaemy M., Berenjestanaki F.R., Bazzar M. (2013). Organosoluble, thermally stable and low dielectric constant fluorinated polyimides containing 2,4,5-triphenylimidazole moiety in the main chains. Des. Monomers Polym..

[7] Xiao Y., Low B.T., Hosseini S.S., Chung T.S., Paul D.R. (2009). The strategies of molecular architecture and modification of polyimide-based membranes for CO_2_ removal from natural gas—A review. Prog. Polym. Sci..

[8] Guan Y., Wang C., Wang D., Dang G., Chen C., Zhou H., Zhao X. (2015). High transparent polyimides containing pyridine and biphenyl units: Synthesis, thermal, mechanical, crystal and optical properties. Polymer.

[9] Sun Z., Liu M., Yi L., Wang Y. (2013). High glass transition of organo-soluble copolyimides derived from a rigid diamine with tert-butyl-substituted triphenylpyridine moiety. RSC Adv..

[10] Roy A., Hickner M.A., Lee H.S., Glass T., Paul M., Badami A., Riffle J.S., McGrath J.E. (2017). States of water in proton exchange membranes: Part A—Influence of chemical structure and composition. Polymer.

[11] Miki M., Horiuchi H., Yamada Y. (2013). Synthesis and gas transport properties of hyperbranched polyimide-silica hybrid/composite membranes. Polymers.

[12] Meng X., Yan J., Fan W., Liu J., Wang Z., Li G. (2014). Thermosetting polyimides and composites based on highly soluble phenylethynyl-terminated isoimide oligomers. RSC Adv..

[13] Shojo D., Yamazaki S., Kimura K. (2015). Hydrothermal synthesis of aromatic polyimide particles by using reaction-induced crystallization. J. Polym. Sci. A.

[14] Tripathi B.P., Shahi V.K. (2011). Organic-inorganic nanocomposite polymer electrolyte membranes for fuel cell applications. Prog. Polym. Sci..

[15] Mechref E., Jabbour J., Calas-Etienne S., Amro K., Mehdi A., Tauk R., Zaouk D., Etienne P. (2016). Synthesis and characterization of a photosensitive organic-inorganic, hybrid positive resin type material: Application to the manufacture of microfluidic devices by laser writing. RSC Adv..

[16] Yao H., Zhang N., Shen K., Song N., Shi K., Zhu S., Zhang Y., Guan S. (2017). From a flexible hyperbranched polyimide to a microporous polyimide network: Microporous architecture and carbon dioxide adsorption. Polymer.

[17] Li J., Zhang H., Liu F., Lai J., Qi H., You X. (2013). A new series of fluorinated alicyclic-functionalized polyimides derivated from natural-(D)-camphor: Synthesis, structure–properties relationships and dynamic dielectric analyses. Polymer.

[18] Sokolova M.P., Smirnov M.A., Geydt P., Bugrov A.N., Ovaska S.S., Lahderanta E., Toikka A.M. (2016). Structure and transport properties of mixed-matrix membranes based on polyimides with ZrO_2_ nanostars. Polymers.

[19] Zhou Y., Chen G., Zhao H., Song L., Fang X. (2015). Synthesis and properties of transparent polyimides derived from trans-1,4-bis(2,3-dicarboxyphenoxy)cyclohexane dianhydride. RSC Adv..

[20] Gao Y., Zhou Y., He M., Wang H., Cui Y., Zhang T. (2014). Synthesis and characterization of fluorinated polyimides derived from 1,4-bis-[4-amino-2-(trifluoromethyl)-phenoxy] benzene/tetrafluoride benzene. Des. Monomers Polym..

[21] Wen P., He R., Li X.D., Lee M.H. (2017). Syntheses and characterizations of high refractive index and low birefringence polyimides containing spirobifluorene in the side chain. Polymer.

[22] Wang F., Shao L.S., Bai Q.Y., Che X., Liu B., Wang Y.H. (2017). Photo-induced vertical alignment of liquid crystals via in situ polymerization initiated by polyimide containing benzophenone. Polymers.

[23] Xia S., Sun Z., Yi L., Wang Y. (2013). Synthesis of soluble polyimide derived from novel naphthalene diamines for liquid crystal alignment layers and a preliminary study on the mechanism of imidization. RSC Adv..

[24] Sun N.W., Meng S.Y., Zhou Z.W., Yao J.N., Du Y.L., Wang D.M., Zhao X.G., Zhou H.W., Chen C.H. (2016). High-contrast electrochromic and electrofluorescent dual-switching materials based on 2-diphenylamine-(9,9-diphenylfluorene)-functionalized semi-aromatic polymers. RSC Adv..

[25] Zhao J., Peng L., Zhu Y.L., Song Y.J., Wang L.J., Shen Y.Z. (2016). Synthesis and memory characteristics of novel soluble polyimides based on asymmetrical diamines containing carbazole. Polymer.

[26] Chen B.K., Wu T.Y., Wong J.M., Chang Y.M., Lee H.F., Huang W.Y., Chen A. (2015). Highly sulfonated diamine synthesized polyimides and protic ionic liquid composite membranes improve PEM conductivity. Polymers.

[27] Van Genabet B., Schwarz A., Bruneel E., Rambausek L., Van Driessche I., Van Langenhove L. (2011). Synthesis and characterization of copper, polyimide and TIPS-pentacene layers for the development of a solution processed fibrous transistor. AIP Adv..

[28] Yu H.C., Jung J.W., Choi J.Y., Chung C.M. (2016). Kinetic study of low-temperature imidization of poly(amic acid)s and preparation of colorless, transparent polyimide films. J. Polym. Sci. A.

[29] Wang X., Li Y., Gong C., Zhang S., Ma T. (2007). Synthesis and characterization of novel soluble pyridine-containing polyimides based on 4-phenyl-2,6-bis 4-(4-aminophenoxy)phenyl-pyridine and various aromatic dianhydrides. J. Appl. Polym. Sci..

[30] Wozniak A.I., Yegorov A.S., Ivanov V.S., Igumnov S.M., Tcarkova K.V. (2015). Recent progress in synthesis of fluorine containing monomers for polyimides. J. Fluor. Chem..

[31] Zhang Y., Shen J., Zhang Q.Y., Xu Z.S., Yeung K.W.K., Yi C.F. (2014). Review on F, Si and P-containing polyimides with special properties. Sci. Adv. Mater..

[32] Dhara M.G., Banerjee S. (2010). Fluorinated high-performance polymers: Poly(arylene ether)s and aromatic polyimides containing trifluoromethyl groups. Prog. Polym. Sci..

[33] Wang C.Y., Cao S.J., Chen W.T., Xu C., Zhao X.Y., Li J., Ren Q. (2017). Synthesis and properties of fluorinated polyimides with multi-bulky pendant groups. RSC Adv..

[34] Liu C.J., Pei X.L., Mei M., Chou G.Q., Huang X.H., Wei C. (2016). Synthesis and characterization of organosoluble, transparent, and hydrophobic fluorinated polyimides derived from 3,3-diisopropyl-4,4-diaminodiphenyl-4-trifluoromethyltoluene. High Perform. Polym..

[35] Lei R., Kang C.Q., Huang Y.J., Qiu X.P., Ji X.I., Xing W., Gao L.X. (2011). Sulfonated polyimides containing pyridine groups as proton exchange membrane materials. Chin. J. Polym. Sci..

[36] Ghaemy M., Khajeh S. (2011). Organosoluble and thermally stable polyimides derived from a new diamine containing bulky-flexible triaryl pyridine pendent group. Chin. J. Polym. Sci..

[37] Mehdipour-Ataei S., Babanzadeh S., Abouzari-Lotf E. (2015). Nicotinic-based poly(amide-ether-imide)s: A new category of soluble, heat-resistant, and flame-retardant polyimides. Des. Monomers Polym..

[38] Huang M., Wang L., Li X., Yan S., Yeung K.W.K., Chu P.K., Xu Z., Yi C. (2012). Design and preparation of novel fluorescent polyimides containingortho-linked units and pyridine moieties. Des. Monomers Polym..

[39] Shen J., Li X., Zhang Y., Wang W., Xu Z., Yeung K.W.K., Xu M., Yi C. (2012). Synthesis and characterization of highly soluble and optically transparent polyimides derived from novel fluorinated pyridine-containing aromatic diamine. High Perform. Polym..

[40] Yan S.Y., Chen W.Q., Yan W., Huang M.F., Chen C., Xu Z.S., Yeung K.W.K., Yi C.F. (2011). Optical transparency and light colour of highly soluble fluorinated polyimides derived from a novel pyridine-containing diamine m,p-3FPAPP and various aromatic dianhydrides. Des. Monomers Polym..

[41] Koohmareh G.A. (2007). New organo-soluble polyimides based on a new dianhydride: 4-(4-t-butyl-phenyl)-2,6-bis(3,4-phenyl dicarboxylic acid anhydride)pyridine. Des. Monomers Polym..

[42] Zhao J.J., Gong C.L., Zhang S.J., Shao Y., Li Y.F. (2010). Synthesis of a new pyridine-containing diamine and related polyimide. Chin. Chem. Lett..

[43] Sadhasivam B., Muthusamy S. (2016). Thermal and dielectric properties of newly developed L-tryptophan-based optically active polyimide and its POSS nanocomposites. Des. Monomers Polym..

[44] Mansoori Y., Sanaei S.S., Zamanloo M.R., Imanzadeh G., Atghia S.V. (2013). Synthesis and properties of new polyimide/clay nanocomposite films. Bull. Mater. Sci..

[45] Wang C.B., Guan Y., Tian D.B., Dang G.D., Wang D.M., Chen C.H., Zhou H.W. (2015). Highly transparent polyimides derived from 2-phenyl-4,6-bis(4-aminophenoxy)pyrimidine and 1,3-bis(5-amino-2-pyridinoxy)benzene: preparation, characterization, and optical properties. RSC Adv..

[46] Li F., Wan W.J., Lai J.C., Liu F., Qi H.X., Li X.S., You X.Z. (2015). Investigations on the polyimides derived from unfunctionalized symmetric cyclopentyl-containing alicyclic cardo-type dianhydride. J. Appl. Polym. Sci..

[47] Bahman T., Gholam A.K. (2007). Synthesis and characterization of new thermally stable poly(ether-imide)s derived from 4-aryl-2,6-bis[4-(3-nitrophthalimido)phenyl] pyridines. Des. Monomers Polym..

[48] An H.Y., Zhan M.S., Wang K. (2009). Synthesis and characterization of soluble poly(ether imide)s containing fluorenyl cardo groups. J. Appl. Polym. Sci..

[49] Huang X.H., Pei X.L., Wang L.C., Mei M., Liu C.J., Wei C. (2016). Design and synthesis of organosoluble and transparent polyimides containing bulky substituents and noncoplanar structures. J. Appl. Polym. Sci..

[50] Xiong L., Wang X., Qi H., Liu F. (2012). Synthesis of a new siloxane-containing alicyclic dianhydride and the derived polyimides with improved solubility and hydrophobicity. J. Appl. Polym. Sci..

