# Supplementary material for: Synthesis and Characterization of Organosoluble, Thermal Stable and Hydrophobic Polyimides Derived from 4-(4-(1-Pyrrolidinyl)phenyl)-2,6-bis(4-(4-aminophenoxy)phenyl)pyridine
Source: Polymers (Basel). 2017 Oct 3;9(10):484. doi: 10.3390/polym9100484 (PMC6418872; doi:10.3390/polym9100484)
Supplement: Supplementary file 1 [file polymers-09-00484-s001.pdf]

Supplementary Materials

Synthesis and characterization of organosoluble, thermal stable and hydrophobic polyimides derived from 4-(4-(1-pyrrolidinyl)phenyl)-2,6-bis(4-(4-aminophenoxy)phenyl) pyridine

Xiaohua Huang *, Beicai Chen, Mei Mei, Hua Li, Chanjuan Liu * and Chun Wei

Key Laboratory of New Processing Technology for Nonferrous Metal & Materials, Ministry of Education, and School of Material Science and Engineering, Guilin University of Technology, Guilin 541004, China

1. Experimental..2

2. Characterization Data...5

3. FT-IR Spectra of compounds...6

4. NMR Spectra of compounds..8

5. MS Spectra of PPAPP..9

1 Experimental

1.1 Characterization Methods

Fourier Transform Infrared (FT-IR) of monomer and polymer were tested on Thermo Nexus 470 FT-IR spectrometer (Thermo Nicolet, USA). ^{1}H NMR and ^{13}C NMR of monomer were measured on an Avance AV 500 instrument (Bruker, Switzerland) Using deuterated dimethylsulfoxide (DMSO-d6) as solvents. Mass spectrometry was recorded on a Elementar Vario EL III/Isoprime (Elementar, Germany). The thermal stability of films was performed on a TGA Q500 analyzer (TA Instruments, Brussels, Belgium). Films were dried at 150 °C for 30 min and then heated from 50 °C to 800 °C at heating rate of 10 °C /min in nitrogen. The glass transition temperatures (T_g) of films was recorded on a DSC-204 phoenix thermal analyzer (Netzsch, Wittelsbacherstr, Germany) at a heating rate of 10 °C /min in nitrogen atmosphere. Solubility analysis: 10 mg of polymer was dissolved in 1 mL organic solvent and observed the dissolving state of polymer at room temperature or heating conditions. Surface hydrophobicity was carried out on the film for water was tested by a contact angle goniometer instrument (JY-PHb, China) with 40% ± 2% relative humidity at the room temperature. A crystallographic study of films was measured at room temperature (about 25 °C) on an X'Pert PRO X-ray diffractometer (PANalytical B.V.). The data was taken from 10o to 40o (2θ values) with Cu/Kα radiation (λ = 0.154 nm, operating at 40 kV and 40 mA). Inherent viscosities of PAA were tested by Ubbelohde viscometer. It was measured at concentration of 0.5 g/dL in NMP at 25 °C.

1.2. Preparation of monomer

Synthesis of 4-(4-nitrophenoxy)acetophenone (NPAP)

In a 500 mL three-necked round bottom flask equipped with magnetic force stirrer, reflux

condenser and nitrogen inlet, 4-hydroxyacetophenone (40.00 g, 0.29 mol), 1-chloro-4-nitrobenzene (46.28 g, 0.29 mol), potassium carbonate (85.27 g, 0.62 mol), and 300 mL DMF was refluxed for 6 h at 145 °C. After the reaction was terminated, the reaction mixture was cooled to 60 °C, and then poured into ice-water with small amount of NaCl. The precipitate was filtered to give yellow product. Finally, the crude product was recrystallized from ethanol and ethyl acetate (V:V=1:1) mixture solution to obtain white pure products 4-(4-nitrophenoxy) acetophenone (NPAP). Yield: 65%.

Synthesis of 4-(4-(1-pyrrolidinyl)phenyl)-2,6-bis(4-(4-nitrophenoxy)phenyl)pyridine (PPNPP)

10 g (0.057 mol) of 4-(1-pyrrolidinyl)benzaldehyde, 29.36 g, (0.11 mol) of NPAP, 52.78 g of ammonium acetate and 130 mL acetic acid were placed into a 500 mL three-necked flask equipped with magnetic force stirrer, reflux condenser and nitrogen inlet. The mixture was refluxed with stirring for 6 h at 125 °C. After the reaction mixture was cooled to room temperature, and then concentrated in a vacuum to remove excess acetic acid, the yellow crude power PPNPP was obtained under vacuum drying at 120 °C for 12 h. The pure PPNPP was acquired after the precipitate purified by recrystallization in DMF and vacuum drier at 120°C. Yield: 61%.

Synthesis of 4-(4-(1-pyrrolidinyl)phenyl)-2,6-bis(4-(4-aminophenoxy)phenyl)pyridine (PPAPP)

To a 500 mL three-necked round-bottomed flask equipped with magnetic force stirrer, reflux condenser and nitrogen inlet, PPNPP (16.62 g, 0.026 mol), Pd/C (3.5 g) and 300 mL of ethanol absolute, then the mixture solution heated to 85 °C for 30 min. After 65 mL hydrazine monohydrate was dropped for 1 h, the reaction mixture was stirred for another 8 h. 80 mL extra tetrahydrofuran was added before the end of the reaction, and then refluxed for another 10 min. The mixture solution was followed by hot filtration to remove excess Pd/C, evaporated and dried

under vacuum at 120 °C for 12 h to get crude product PPAPP. The pure PPAPP was obtained by silica gel column chromatography ($V_{Dichloromethane}$:$V_{Ethyl\ acetate}$ = 30:2, Rf = 0.35) and vacuum drier at 120 °C. Yield: 90%.

1.3. Preparation of polyimides

The polyimides were synthesized from PPAPP diamines and various dianhydrides *via* two-step method. A typical example of polymerization is as follows. The synthesis of PI-1 (PPAPP and PMDA) is used as an example to illustrate the general synthetic route used to produce the polyimides. A mixture of solution of 0.6176 g (0.001042 mol) PPAPP and 5 ml of dry NMP in 25 ml flask, 0.2273 g (0.001042 mol) of dianhydride PMDA was added in two portions. Therefore, the solid content of the solution was maintained approximately 20 wt%. The mixture was stirred at room temperature for 24 h to afford highly viscous poly(amic acid)s (PAA) solution. The PAA was subsequently converted into polyimide by the thermal imidization process. For the process, the PAA solution was poured onto a clean glass substrate, and then placed at 80 ^{o}C for 12 h to slowly to release the most casting solvent. The semidry PAA was further dried by programmed heating at 150 ^{o}C, 200 ^{o}C, 250 ^{o}C and 300 ^{o}C for each 1 h, and then the PI film was peeled off from the glass substrate by immersion in water. Finally, PI-1 (PPAPP-PMDA) film was obtained after drying at 150 °C for 12 hours before being measured. Simultaneously, the other PAA solutions were prepared by similar procedure, and the thermal imidization method was also adopted by the above method to obtained other PI films, which named PI-2 (PPAPP-BPDA), PI-3 (PPAPP-ODPA), PI-4 (PPAPP-BTDA), and PI-5 (PPAPP-6FDA).

2. Characterization Data

Synthesis of 4-(4-nitrophenoxy)acetophenone (NPAP)

^{1}H NMR (500 MHz, DMSO, δ, ppm): 8.25 (d, 2H), 8.04 (d, 2H *J* = 8.4 Hz), 7.22 (t, 4H, *J* = 9.8 Hz), 2.57 (s, 3H, $-CH_3$). ^{13}C NMR (500 MHz, DMSO, δ, ppm): 197.32, 162.14, 159.27, 143.69, 134.19, 131.65, 126.97, 120.31, 119.38, 27.39. FT-IR (KBr, cm^{-1}): 3109, 3073 ($-CH_3$), 1681 (C=O), 1507, 1347 ($-NO_2$), 1168 (C-O-C). m.p. 83 °C (by DSC at a scan rate of 5 K/min).

Synthesis of 4-(4-(pyrrolidinyl)phenyl)-2,6-bis(4-(4-nitrophenoxy)phenyl)pyridine (PPNPP)

^{1}H NMR (500 MHz, DMSO, δ, ppm): 8.46 (d, *J* = 8.7 Hz, 4H), 8.31 (d, *J* = 9.0 Hz, 4H), 8.17 (s, 2H), 7.98 (d, *J* = 8.8 Hz, 2H), 7.36 (d, *J* = 8.7 Hz, 4H), 7.25 (d, *J* = 9.5 Hz, 2H), 6.69 (d, *J* = 8.9 Hz, 2H), 3.35 (d, *J* = 24.5, 6H), 2.02 (m, 4H). FT-IR (KBr, cm^{-1}): 1596, 1439 (pyridine), 1514, 1342 ($-NO_2$), 3100 ~ 3000 (pyrrolidine), 1165 (C-O-C). m.p. 267 °C (by DSC at a scan rate of 5 K/min).

Synthesis of 4-(4-(pyrrolidinyl)phenyl)-2,6-bis(4-(4-aminophenoxy)phenyl)pyridine (PPAPP)

^{1}H NMR (500 MHz, DMSO, δ, ppm): 8.23 (d, 4H, *J* = 8.5 Hz), 7.95 (s, 2H), 7.87 (d, 2H, *J* = 8.5 Hz), 7.00 (d, 4H, *J* = 8.5 Hz), 6.87 (d, 4H, *J* = 8.5 Hz), 6.65 (m, 6H), 5.05 (s, 4H, $-NH_2$), 3.28 (t, 4H, *J* = 6 Hz), 1.96 (m, 4H,). ^{13}C NMR (500 MHz, DMSO, δ, ppm) 160.24, 156.15, 149.77, 148.84, 145.90, 145.82 , 133.40, 128.81, 128.31, 121.48, 116.85, 115.53, 114.11, 112.33, 47.73, 43.45, 25.43. FT-IR (KBr, cm^{-1}): 3300 ~ 3500 ($-NH_2$), 3100 ~ 3000 (pyrrolidine), 1168 (C-O-C), 1595, 1438 (pyridine). MS (m/z): 591.27 ($[M+H]^+$). m.p. 181 °C (by DSC at a scan rate of 5 K/min).

3. FT-IR Spectra of compounds

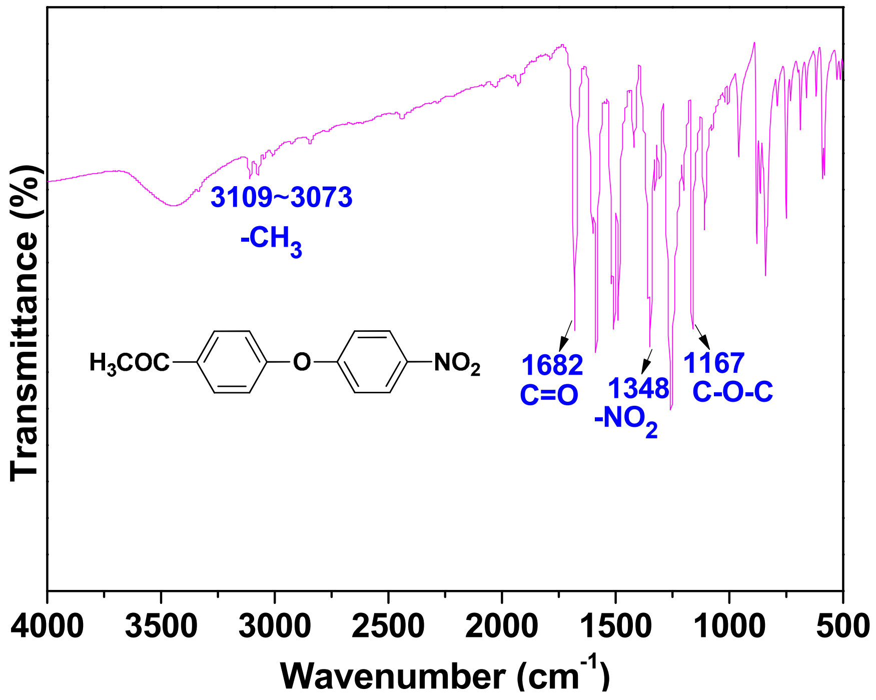

Figure S1. FT-IR spectra of compound NPAP

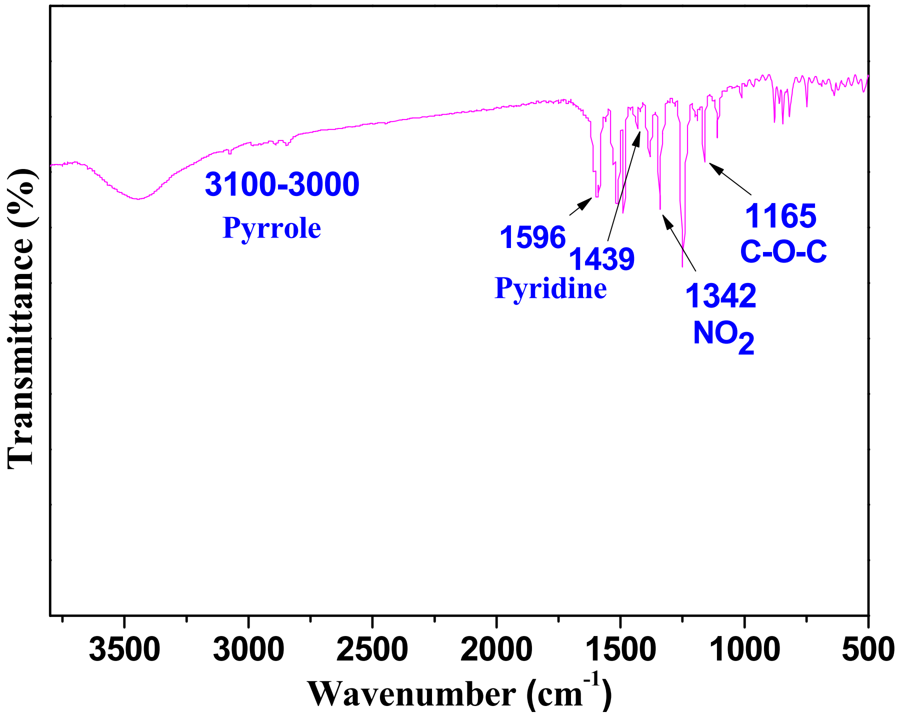

Figure S2. FT-IR spectra of compound PPNPP

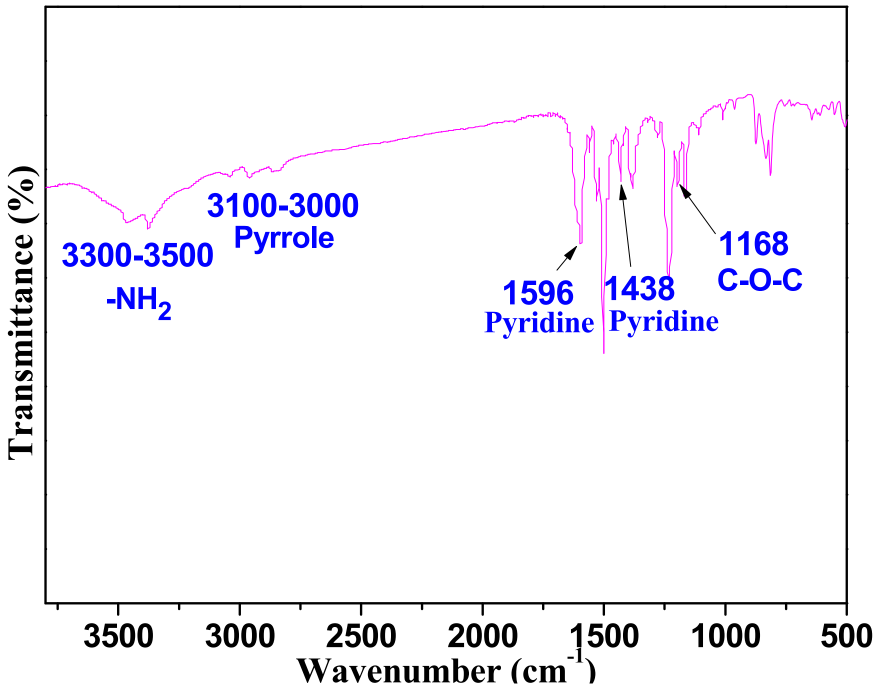

Figure S3. FT-IR spectra of diamine PPAPP

4. NMR Spectra of compounds

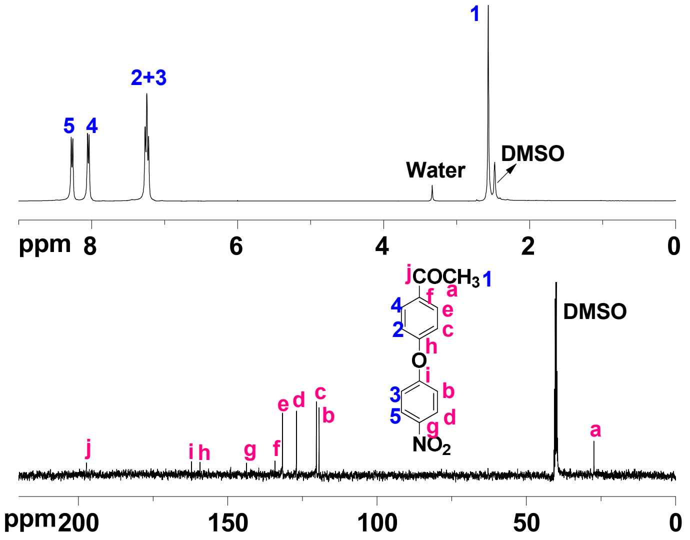

Figure S4 ^{1}H and ^{13}C NMR spectra of compound NPAP

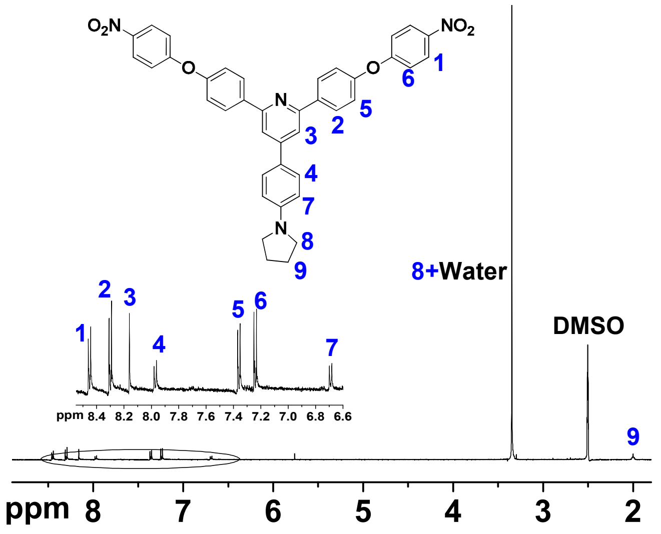

Figure S5 ^{1}H-NMR spectra of compound PPNPP

5. MS Spectra of PPAPP

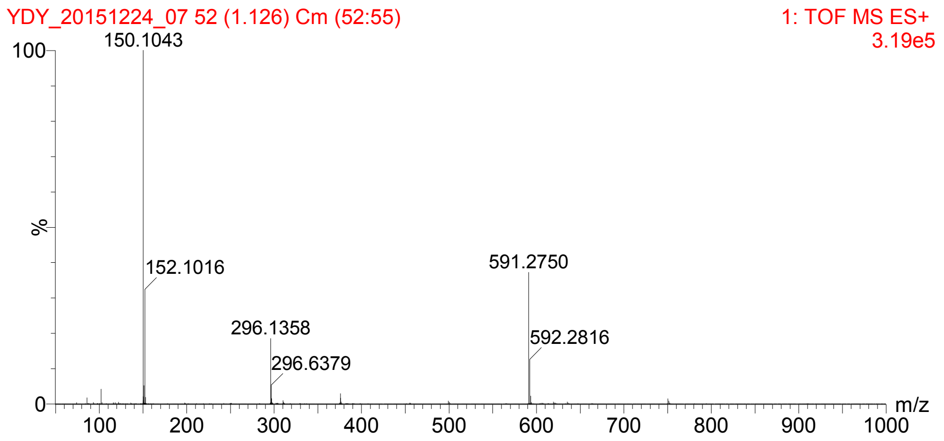

Figure S6. MS spectra of diamine PPAPP
